# COMBINED HEART-LIVER-KIDNEY TRANSPLANTATION: THE FIRST EXPERIENCE IN LATIN AMERICA

**DOI:** 10.1590/0102-672020210002e1668

**Published:** 2022-06-24

**Authors:** Eduardo FERNANDES, Filipe Roza da SILVA, Rodrigo SEGALOTE, Pedro Túlio ROCHA, Felipe MELLO, Camila GIRÃO, Ronaldo OLIVEIRA, Leandro SAVATTONE, Camila CESAR, Munique SIQUEIRA, Adolpho BAAMONDE, Gabrielle OLIVEIRA, Rodrigo FURTADO, Camila TOBIAS, Thays RIBEIRO, Carlos GALHARDO, Gabriel MOURAD, Felipe ROBALINHO, Anderson BRITO

**Affiliations:** 1 Heart Transplant Unit, Hospital São Lucas de Copacabana, Rio de Janeiro - RJ, Brazil;; 2 Liver Transplant Unit, Hospital São Lucas de Copacabana, Rio de Janeiro - RJ, Brazil;; 3 Kidney Transplant Unit, Hospital São Lucas de Copacabana, Rio de Janeiro - RJ, Brazil;; 4 Hospital São Lucas de Copacabana, Rio de Janeiro - RJ, Brazil.

**Keywords:** Heart Transplantation, Liver Transplantation, Kidney Transplantation, Transplants., Transplante Cardíaco, Transplante Hepático, Transplante Renal, Transplantes.

## INTRODUCTION

Multiorgan dysfunction involving the liver and kidneys is common in patients with end-stage heart failure. The first combined liver-kidney transplant (CLKT) was performed by Margreiter in 1983, and it is a routine procedure in many transplant centers representing 1-8% of liver transplant candidates^5^.

Hepatic and renal dysfunction may be secondary to a systemic disease process affecting the heart, liver, and kidneys or may be a consequence of heart failure with venous congestion and arterial hypoperfusion^2^ ^,^ ^4^.

Despite the complexity and costs involved, the results of this type of transplant are optimistic, as these are terminally ill patients.

Our objective was to report the first Latin American experience in triple transplantation in the same surgical time (heart, liver, and kidney), which took place at Hospital São Lucas Copacabana^1^.

### Case Report

The organ receiver was a 56-year-old male, physical education teacher, diagnosed with dilated cardiomyopathy with biventricular dysfunction of undetermined origin and compensated cirrhosis of probable cardiogenic etiology diagnosed in 2018 during a cholecystectomy. Chronic kidney disease was due to cardiorenal syndrome.

After follow-up of the case, triple liver-cardiac-renal transplantation was indicated.

**Figure 1 -:**
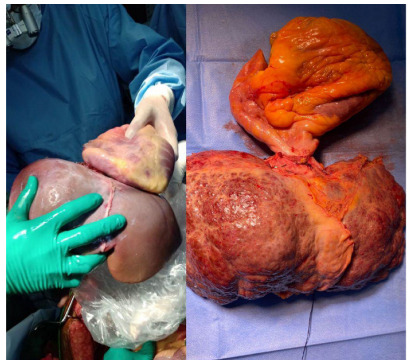
Donor organs on the left and organs from the receiver to the right.

The patient was registered in the national transplant system according to the MELD scale. He remained on the transplant waiting list for 17 days. An 18-year-old, a victim of polytrauma, donor was available. The organ harvesting took place on the same day (Figure 1).

**Figure 2 -:**
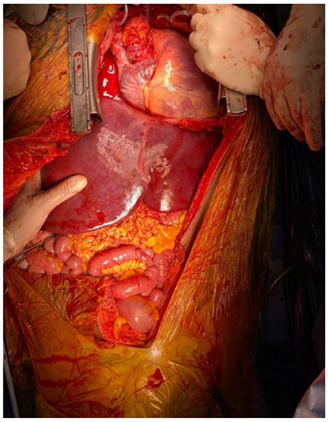
Organs already implanted in the receiver.

*En bloc* technique was used with a single piece consisting of the heart and liver and the kidneys were removed separately. The implant took place on April 5, 2022 and lasted for 8 h, with the heart implant performed by the cardiothoracic surgery team, and the liver and kidney implant performed by the abdominal surgery team (Figure 2).

The left femoral vein was cannulated and positioned below the renal veins, followed by cannulation of the superior vena cava and ascending aorta with subsequent initiation of cardiopulmonary bypass. For transplantation, the anterior diaphragm was opened up to the inferior vena cava with removal of the piece *en bloc* (heart *plus* liver) and subsequent insertion of the graft also *en bloc*. Later, a bicaval orthotopic heart transplant was performed.

For liver transplantation, hepatectomy was performed using the cross-clamp technique. The implantation took place with the completion of end-to-end anastomosis of the infrahepatic vena cava, end-to-end anastomosis of the portal vein, and end-to-end anastomosis of the hepatic artery.

Then, renal implantation was performed with end-to-side anastomoses of the renal vein of the donor’s right kidney with the recipient’s right common iliac vein. End-to-side anastomosis of the renal artery of the right kidney of the donor with the right internal iliac artery of the recipient was performed, followed by implantation of the ureter by the extravesical technique. After renal reperfusion, a bile duct anastomosis was performed.

The patient was referred to the ICU, where he remained for 6 days, and discharged after 11 days.

## DISCUSSION

Combined heart-liver-kidney transplantation is quite rare, with few cases reported in the literature so far. Our case report represents the first transplant of its kind performed in Latin America. In this case, an atypical preparation was required involving different teams of surgeons, in addition to dozens of professionals responsible for logistics.

The *en bloc* technique was performed, in which the liver and heart remain connected by the inferior vena cava. It was possible to perform the cardiac and hepatic implant simultaneously. One of the benefits of this technique is that it allows the heart and liver to be perfused simultaneously, minimizing cold hepatic ischemia time. In addition, the patient is placed on cardiopulmonary bypass, which facilitates not only cardiac but also hepatic implantation, reducing the hemodynamic impact on the liver implant and assisting in blood oxygenation so that the grafts can later recover from the period of ischemia^1^.

Ebong et al ^2^ showed that the presence of advanced liver or kidney disease may increase the likelihood of complications and unfavorable outcomes after heart transplantation if liver and kidney transplantation are not performed concomitantly.

Another benefit associated with combined organ transplantation was evidenced by Ortega-Legaspi et al who showed immunological protection provided by a liver allograft from the same donor in cases of multiple organ transplantation^2^ ^,^ ^3^ ^,^ ^4^.

The choice of the *en bloc* technique also reduces the surgical time as it reduces the number of anastomoses required. In our case, after the heart-liver implant, renal implantation was performed. This choice was made as a strategy if there was any intercurrence or instability during organ implantation. The renal graft allows longer ischemia times and could be implanted within 24 h, after hemodynamic stabilization of the patient, if necessary^1^.
